# Experimental Study and FEM Simulations for Detection of Rebars in Concrete Slabs by Coplanar Capacitive Sensing Technique

**DOI:** 10.3390/s22145400

**Published:** 2022-07-20

**Authors:** Farima Abdollahi-Mamoudan, Clemente Ibarra-Castanedo, Tobin Filleter, Xavier P. V. Maldague

**Affiliations:** 1 Department of Electrical and Computer Engineering, Université Laval, 1065 Avenue de la Médecine, Québec, QC G1V 0A6, Canada; xavier.maldague@gel.ulaval.ca; 2 Department of Mechanical and Industrial Engineering, University of Toronto, 5 King’s College, Toronto, ON M5S 3G8, Canada; filleter@mie.utoronto.ca

**Keywords:** coplanar capacitive technique, reinforced concrete (RC), NDT methods, finite element modelling (FEM), capacitive sensor

## Abstract

In the present study, a relatively novel non-destructive testing (NDT) method called the coplanar capacitive sensing technique was applied in order to detect different sizes of rebars in a reinforced concrete (RC) structure. This technique effectively detects changes in the dielectric properties during scanning in various sections of concrete with and without rebars. Numerical simulations were carried out by three-dimensional (3D) finite element modelling (FEM) in COMSOL Multiphysics software to analyse the impact of the presence of rebars on the electric field generated by the coplanar capacitive probe. In addition, the effect of the presence of a surface defect on the rebar embedded in the concrete slab was demonstrated by the same software for the first time. Experiments were performed on a concrete slab containing rebars, and were compared with FEM results. The results showed that there is a good qualitative agreement between the numerical simulations and experimental results.

## 1. Introduction

Reinforced concrete (RC) has been the most commonly used building material in civil engineering structures for over a century because it is cost-effective, durable and can be cast in any shape. The distribution of rebars inside RC structures is controlled by design and is carefully selected to provide the necessary strength to withstand all loads applied to the RC elements [1,2]. Sometimes, knowing the location of the rebars in the RC structures (usually, after the concrete has hardened) is important to analyse the quality of new elements and/or to verify the condition of existing structures for which design details are lacking [3]. Parameters such as a poor design and harsh environment can combine to cause damage to a concrete structure. This may lead to the structure failing by creating visually unacceptable surface cracks, and may eventually cause further corrosion and structural failure [2]. Most of the time, this deterioration may not emerge on the surface of the structures until catastrophic damages have happened. Therefore, the use of non-destructive testing (NDT) techniques is critical for evaluating the condition of concrete structures and preventing failures. In recent years, innovative NDT methods that can be used to evaluate existing structures have become available to detect rebars in reinforced concrete, and possess their own advantages and limitations [4].

Ultrasonic techniques have been employed as an NDT method to evaluate concrete structures [5] and for inspecting the rebars through the use of a variety of transducer technologies and frequencies [6]. However, ultrasonic techniques often encounter the problem of scattering, in which, the ultrasonic wave may bypass the rebar, causing uncertainty about the measurement data [7]. Radiography is another NDT method to inspect RC structures to acquire information on the quality of concrete and its defects. It is found that this technique is a reliable method for inspecting internal cracks and cavities. However, its radiation exposure is dangerous, and more precautions should be taken [8]. Ground penetrating radar (GPR) is the widely used technique for recognizing the location and depth of buried rebars in RC structures [9,10]. Although this technique has a significant penetration depth [11], it is specified that it has a low accuracy and that the measured data require intensive processing for interpretation [1].

Recently, the coplanar capacitive sensing technique has been employed as an NDT method to inspect and identify rebars in concrete structures [12]. This technique is also used for the inspection of composites [13] and ceramic tiles [14] due to its low cost [15], non-contact detection, no radiation and high-efficiency level (fast and accurate response) [7]. In addition, there is no need to prepare the surface of the specimen with water or gel as a couplant to perform the inspection [16]. Furthermore, the coplanar capacitive sensing method can be applied to image the internal structure in non-conducting materials and detect flaws in insulator–metal hybrid structures, which shows the capability of the technique for inspecting corrosion under insulation (CUI) [17,18].

The coplanar capacitive sensing technique is an NDT electromagnetic method that produces an electric field distribution when an alternating current (AC) voltage is applied between its main electrodes. Scanning the probe on a specimen changes the electric field distribution, causing a variation in the output voltage [16]. The behaviour of this technique is like a regular capacitor, and the difference is placing the electrodes coplanar (placed next to one another) instead of placing them parallel [19]. As a result, the probe provides single-sided access to specimens during the inspection, which is an important advantage that is especially useful when access to both sides of the sample is restricted. Furthermore, in this technique, the lift-off (the air gap between the surface of the sample and electrodes) can be adjusted according to the problem at hand, making it possible to investigate difficult applications, such as the detection of corrosion under insulation [20]. These advantages of the coplanar capacitive technique make it a potential method in NDT.

In this paper, the potential and feasibility of a coplanar capacitive probe, which owns certain advantages over traditional NDT methods, was investigated in order to inspect an RC structure to detect the rebars and recognise their various sizes. Therefore, a three-dimensional (3D) finite element modelling (FEM) simulation in COMSOL™ Multiphysics software was performed to simulate the electric field distribution from a coplanar capacitive probe to understand the impact of the presence of rebars on the field pattern, and, hence, on the probe output, in comparison to a sound (without rebars) concrete specimen. Afterwards, the 3D FEM simulation was developed and another model was constructed to show the surface flaws at different positions on the rebar. These simulations, which have not been indicated in the previous works, can be considered as a simulation of corrosion on the rebar surface, affect the electric field distribution and lead to a field perturbation. In addition, the real specimen, a concrete slab composed of different sizes of rebars, was simulated using FEM, and the electric field strength for this sample was measured. A rectangular coplanar capacitive sensor was manufactured by etching a printed circuit board (PCB) method, and physical experiments were carried out to inspect a concrete slab composed of various sizes of rebars with the mentioned probe to validate the accuracy of the numerical simulation. The analysis and the synthesis of the results are presented in the following sections.

## 2. Materials and Methods

In a coplanar capacitive sensor, the geometry of the electrodes is an important factor, since it determines the electric field strength and penetration depth, and, hence, the probe performance [21]. In the concentric geometry, the size of the driving and sensing electrodes are not equal, and the output does not depend on the scan direction. The coupling between the driving and sensing electrodes enhances with smaller spacing between the two closest edges of the driving and sensing electrodes for a triangular/rectangular electrode shape. However, the rectangle electrode shape is favoured over the other shapes in this paper since the electric field strength is slightly higher in comparison to the triangle shape, and, in particular, they are suggested when there is a restriction on the overall size of the coplanar capacitive sensor [22], especially when a multi-electrode structure is employed [23].

**Figure 1:**
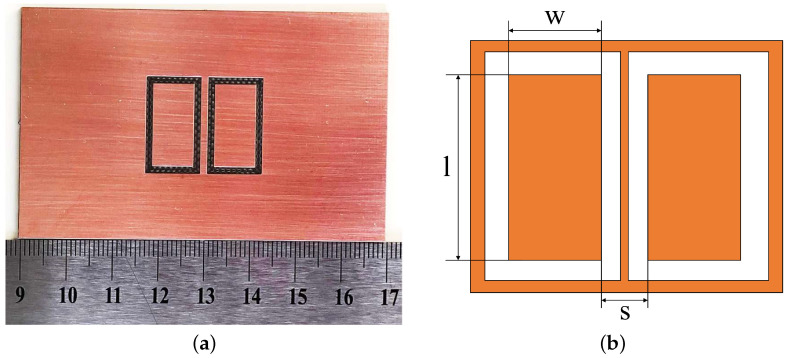
(**a**) Photograph of the rectangular coplanar capacitive probe used in this paper and (**b**) schematic diagram of a rectangular probe. *l*, *w* and *s* represent the length, width and separation distance, respectively.

Figure 1 shows the coplanar capacitive probe used in this work. This probe consists of rectangular electrodes with the same overall size, with *l* = 18 mm (length), *w* = 9 mm (width) and *s* = 4.5 (separation distance between the closest points of the two electrodes). Further, a guard electrode was designed to surround the coplanar rectangular electrodes and between them, which causes shaping of the electric field lines, and a shielding plate was also placed on top of the electrode to remove the ambient noise and to block unwanted electric fields [24]. Both the guard electrode and shielding plate cause an increase in the depth of penetration of the electric field [22].

Numerical simulation, finite element modelling (FEM) and physical experiments were employed to demonstrate the potential of the coplanar capacitive sensing technique for inspecting reinforcement concrete, which are fully described in the following sections.

## 3. Finite Element Modelling (FEM) of an RC Structure

### 3.1. Simulation Setup

The FEM model is a useful tool for predicting the behaviour of the NDT methods [25,26], and especially for predicting the electric field distribution generated from the coplanar capacitive probe. Therefore, a three-dimensional (3D) finite element model was used to analyse the electric field distribution generated by the coplanar electrodes and to identify how rebars in a concrete slab affected this electric field pattern. Numerical simulations were performed by the AC/DC module, an electric currents (ec)-type of COMSOL™ Multiphysics FE package in the 3D domain that can be employed to model the electric field distribution under various conditions.

**Figure 2:**
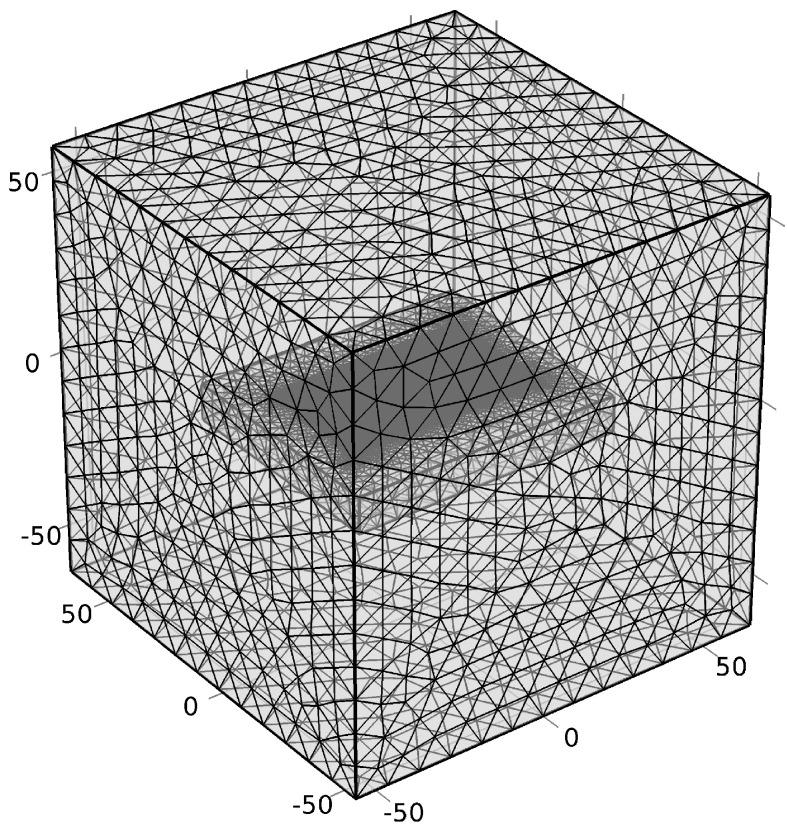
The model after finite element mesh generation and the mesh used to discretise the domain.

**Figure 3:**
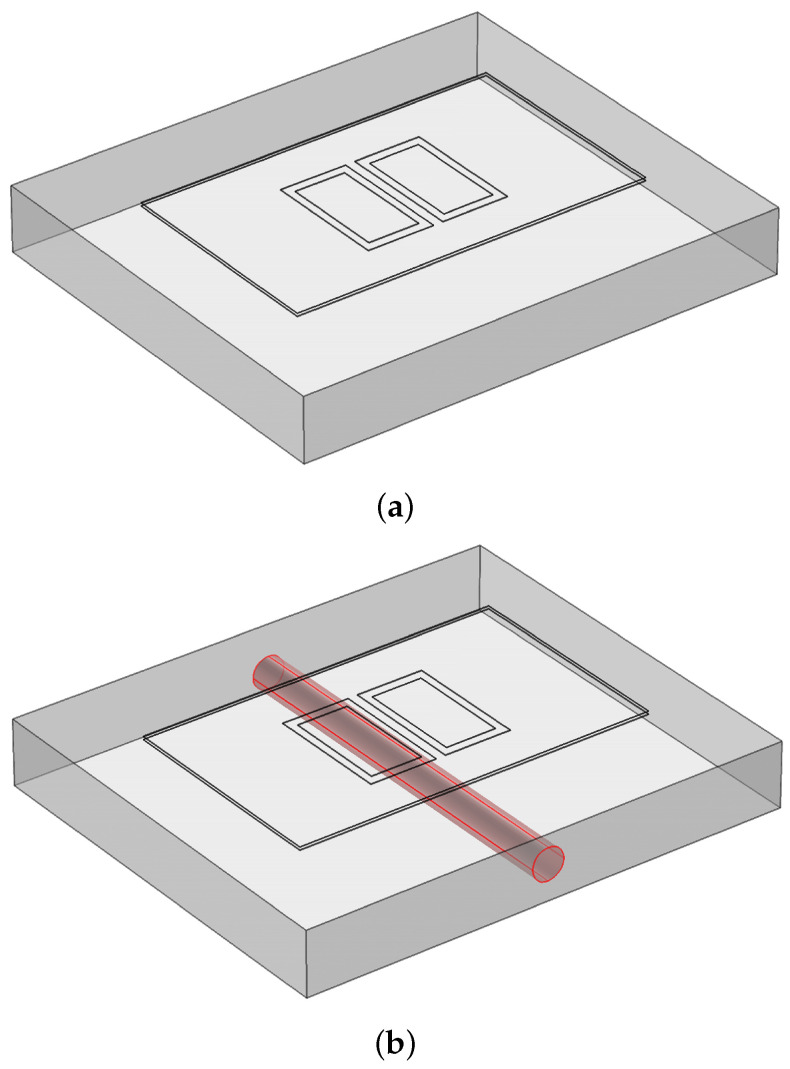
Model geometry for a concrete slab (**a**) without and (**b**) with rebar.

A pair of the rectangular coplanar capacitive electrode with a shielding plate and guard electrode, as shown in Figure 1, was employed in this model. The driving and sensing electrodes were excited by a +10 and −10 V voltage, respectively, at a 100 kHz sinusoidal signal. A zero-charge boundary airbox of approximately three times the size of the region of interest was defined as the simulation domain. A sample was located in the middle of the airbox and the probe was placed above the surface of a concrete slab, at a 1 mm lift-off (the air gap between the surface of the sample and probe). A ground potential was considered for the guarding and shielding. The setup was enclosed in a 110 mm × 110 mm × 110 mm air-filled block centred at the point (*x* = 0, *y* = 0, *z* = 0) as a computational domain. A user-controlled mesh was used and the mesh generation density was set to “Fine” for the airbox and “Finer” for the specimen and probe. Figure 2 shows the model after finite element mesh generation and the mesh used to discretise the domain. The summary of these model specifications is indicated in Table 1. Based on this, an electric field was generated between the driving and sensing electrodes that can penetrate the specimen. Two types of specimens, concrete slabs with/without rebar, as shown in Figure 3, were created to study the electric field distribution in these slabs. Figure 3b presents the model for a concrete slab containing rebar.

### 3.2. FEM Results

**Figure 4:**
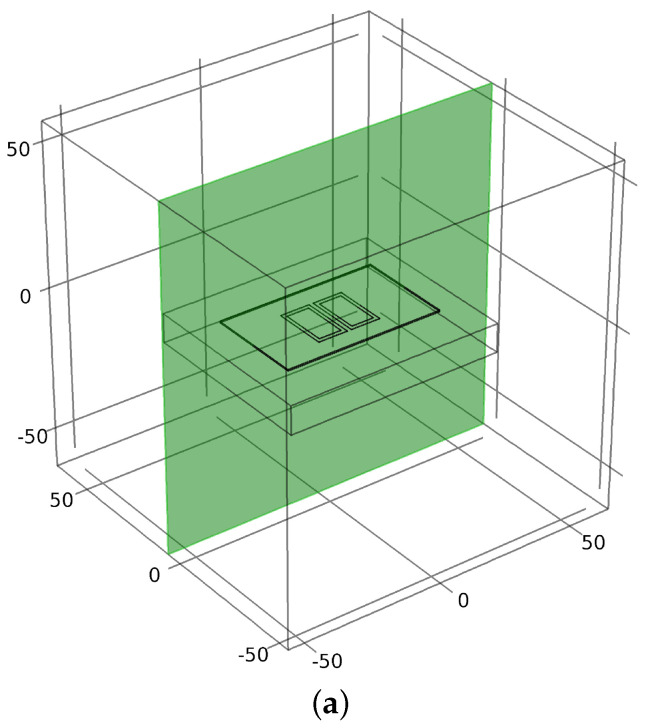

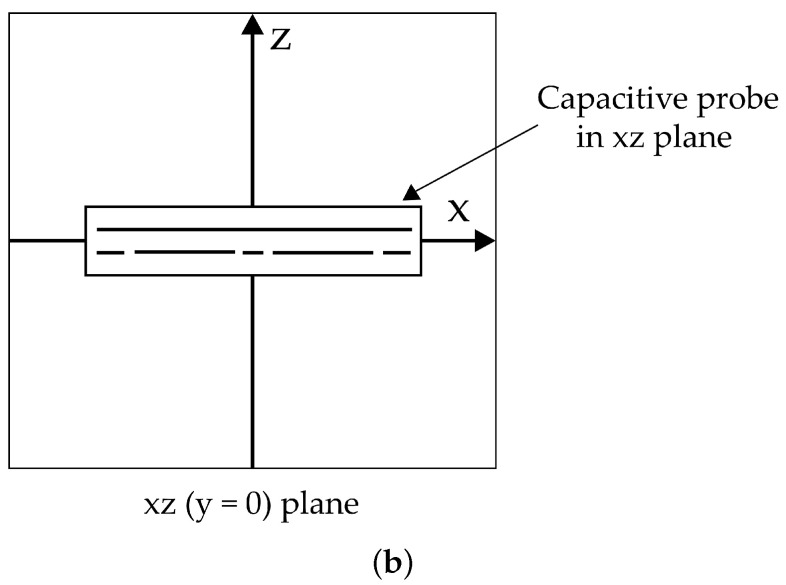
(**a**) *xz* (*y* = 0) plane along the long axis of the capacitive probe symmetry, and (**b**) schematic plane coordinate system for *xz* cross-section.

The numerical simulation results are presented in the *xz* plane (*y* = 0 plane). Figure 4 demonstrates the coordinate system in which the probe surface is centred at the *xz* plane, and the simulation results will be illustrated based on this. The *xz* plane (or *y* = 0 plane) is the cross-sectional plane along the long axis of the probe symmetry, as presented in Figure 4a,b, which show the schematic result for the plane coordinate system for the cross-section.

**Figure 5:**
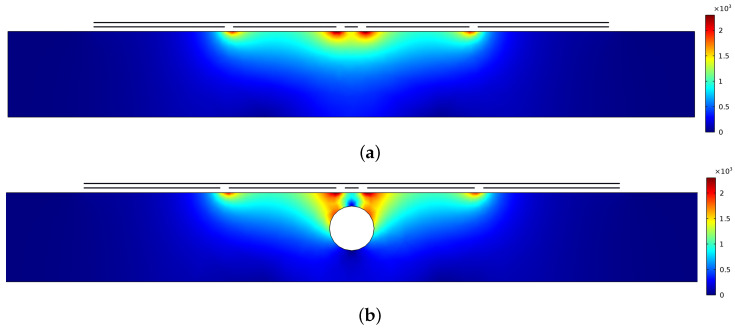
Surface plot of the electric field distribution (V/m) for a concrete slab in *xz* plane: (**a**) sound sample, (**b**) a concrete slab with rebar.

A surface plot of the electric field distribution generated from the coplanar probe in the *xz* plane is demonstrated in Figure 5 for (a) a sound concrete slab and (b) a concrete slab with a rebar. In the case of the concrete slab with an internal rebar (Figure 5b), it is observed that the electric field penetrates the concrete slab and reaches the surface of the conductive rebar, therefore affecting the pattern of the electric field and, hence, the output signal. Accordingly, the coplanar capacitive probe, which has its own certain advantages, can be employed for detecting rebars in RC structures.

**Figure 6:**
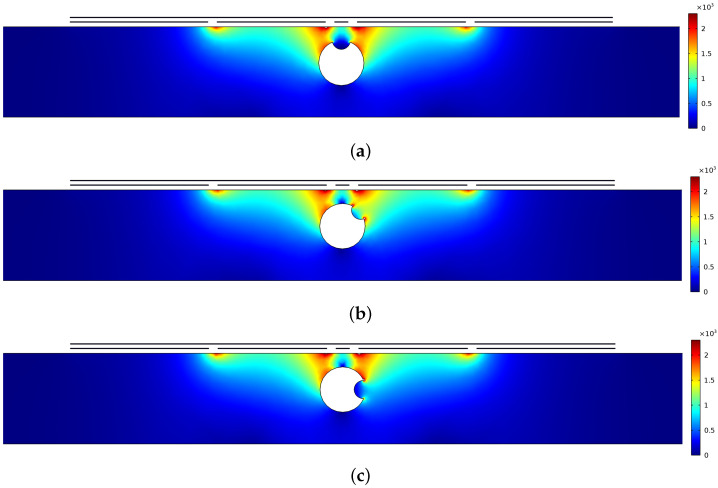
Simulations of the electric field distribution (V/m) in the *xz* plane for a concrete slab with rebar. The rebar contains surface defect at different positions: (**a**) 0°, (**b**) 45° and (**c**) 90° with respect to the electrodes plane.

Another set of simulations was carried out with a rebar containing surface defects (in three different positions) in a concrete slab to demonstrate the distortion of the electric field due to this defect (simulated corrosion). Figure 6 illustrates a surface plot of the electric field distribution produced by the capacitive probe for this specimen in the *xz* plane. From the simulation results, it is obvious that the electric field penetrates the concrete slab and is impacted by the flaw in the rebar surface; therefore, this defect influenced the field pattern, causing a variation in the electric field distribution.

From these results of the 3D FEM numerical simulation for the electric field distribution of a coplanar capacitive probe, the variations in the pattern of the electric field in a sound non-conducting specimen (concrete slab) and how this pattern can change with placing the rebar in the concrete slab can be observed. In addition, based on the second set of the numerical simulation, it can be concluded that the coplanar capacitive probe is sensitive to the surface feature of the conducting material caused by the presence of simulated corrosion on the rebar surface, and that this will lead to a detectable variation in the output signal, and, hence, can be employed for detecting surface flaws on rebars in the RC structure. Therefore, it can be inferred that the technique is a promising method to inspect such structures.

**Figure 7:**
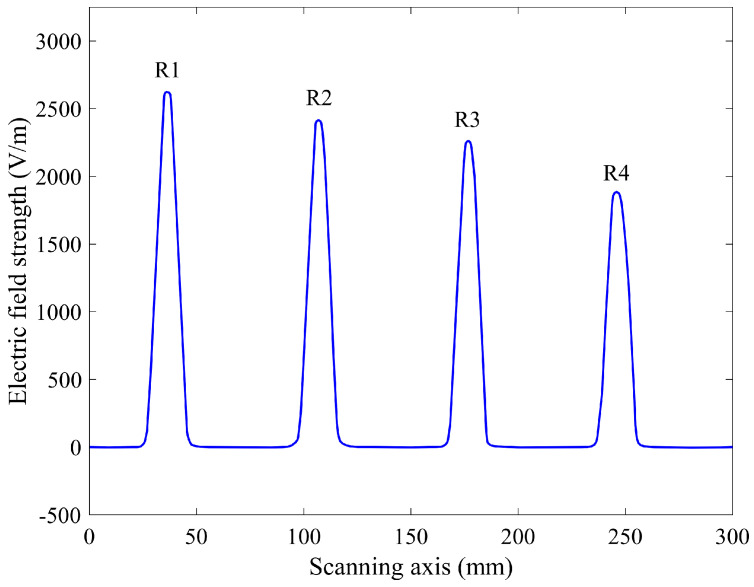
FEM result showing the electric field strength (V/m) of the coplanar capacitive probe inspecting a concrete slab composed of various diameters of rebars. Rebar’s diameters are 5/8, 4/8, 3/8 and 5/16 inches, and their names were abbreviated as R1, R2, R3 and R4 from left to right, respectively.

In addition, some simulations were performed to indicate a comparison between the experimental results and the simulation results. The FEM model of the coplanar capacitive probe inspecting a concrete slab composed of various sizes of rebars is shown in Figure 7. The rebars were placed at a depth of 10 mm; their diameter was 5/8, 4/8, 3/8 and 5/16 inches, and their name was abbreviated as R1, R2, R3 and R4 from left to right, respectively. Figure 7 indicates the scan result of this RC structure with the simulation. From this result, which has also been validated by a physical experiment, it is clear that the probe could detect the rebars, and that the electric field strength decreases when decreasing the diameter of the rebar. Consequently, a coplanar capacitive sensor can constitute an interesting alternative to some other NDT methods with some inherent drawbacks.

## 4. Physical Experiment

### 4.1. Experimental Setup

To verify the accuracy of FEM simulations, a set of physical experiments was also conducted on reinforcement concrete. A pair of coplanar rectangular electrodes, the same probe as shown in Figure 1, was used to inspect the RC structure and detect the rebars. The sensor also included a shielding plate and guard electrode to improve the probe performance. In this paper, the coplanar rectangular electrodes and the guard electrode were manufactured by etching a printed circuit board (PCB) substrate and were coated in copper on its back surface, which is a shielding plate.

**Figure 8:**
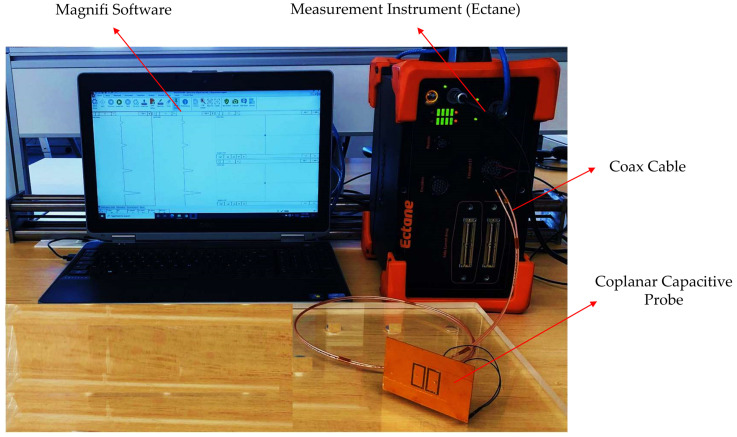
The experimental setup employed for inspecting the RC structure.

Each of the main electrodes can be used as either a driving or a receiving electrode. To induce an electric field, one of the plates was used as a transmitter and connected to the input pin of an Eddyfi Ectane® instrument, and excited by a 10 V amplitude voltage at 100 kHz frequency. The other electrode was considered as the sensing electrode and connected to the output pin of the Ectane®. The shielding plate and guard electrode were connected to the ground pin. To minimise the effect of the noise from cabling on the output signal, the capacitive probe was connected to Ectane® with a coax wire. There is a differential amplifier in Ectane® for consecutively processing the received signal, and its output signal can be a DC voltage. Ectane® is connected to the computer by a cable and the data are sent to Magnifi, which is a specific software designed for Ectane® to process the received signals. Figure 8 indicates the experiment setup employed for the inspecting of an RC structure presented in this paper.

**Figure 9:**
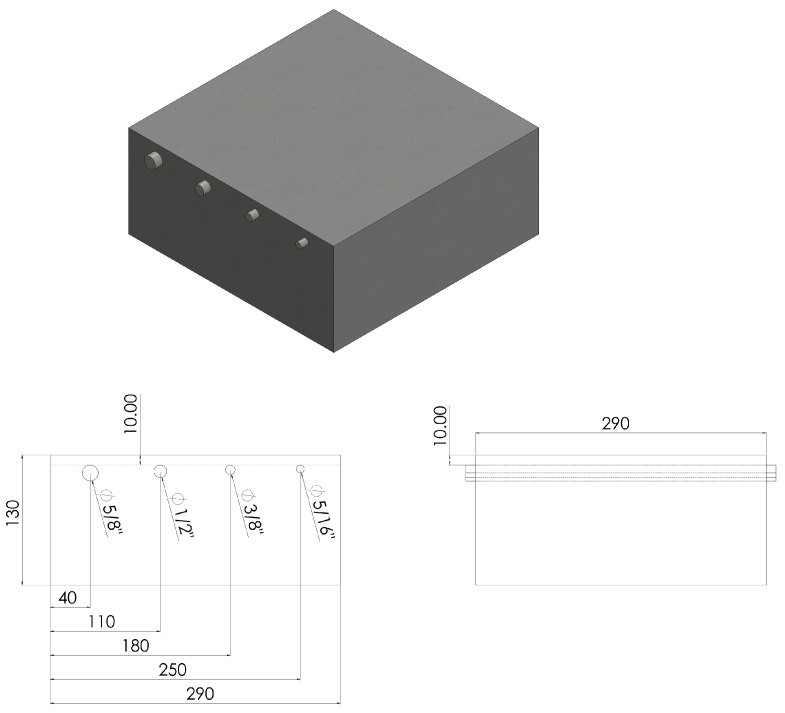
Schematic diagram of a concrete sample with rebars. The diameter of the rebars is 5/8, 4/8, 3/8 and 5/16 inches from left to right, respectively. They are placed at a distance of 10 mm below the concrete surface.

A set of experiments was designed and conducted to evaluate the performance of the coplanar capacitive probe on a concrete slab to inspect the rebars. Figure 9 shows the schematic diagram of the reinforcement concrete specimen. The specimen investigated was a 290 mm × 200 mm × 130 mm concrete slab composed of various diameters of rebars. The standard diameter rebars were embedded within the specimen, at a distance of 10 mm below the concrete surface. The diameters of the rebars were 5/8, 4/8, 3/8 and 5/16 inches from left to right, respectively. The distance between any centre of two adjacent rebars was 63 mm. The coplanar capacitive probe was placed upon the concrete surface at a lift-off of 1 mm.

### 4.2. Experimental Result

**Figure 10:**
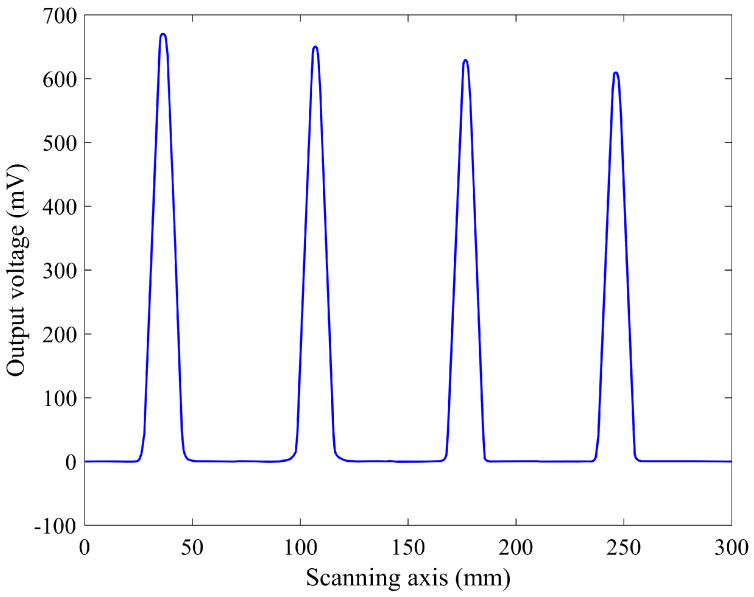
Experimental result showing the output voltage (mV) for various diameters of rebars placed at a depth of 10 mm. The diameter of the rebars is 5/8, 4/8, 3/8 and 5/16 inches from left to right, respectively.

The performance of the coplanar capacitive sensor is based on detecting variations in the electrical properties of the test material, and the characteristic structure of the concrete slab is achieved by scanning the sensor across the surface of the sample. The coplanar capacitive probe was scanned on the sample at a 1 mm lift-off and the results are indicated in Figure 10, which illustrates the line scan results for the rebars located at a depth of 10 mm with different diameters.

From Figure 10, it is obvious that the coplanar capacitive probe could detect the rebars in the concrete slab successfully. This is due to the fact that the capacitive sensor is sensitive to changes in material properties. In fact, the variation in electrical properties between the concrete and rebar affects the electric field pattern and, consequently, the detectable signal on the sensing electrode. It can also be observed that the coplanar capacitive probe easily detected the difference between the size of the rebars so that the rebar with a larger diameter has the greater output while the output voltage diminishes as the rebar diameter is reduced. Therefore, the existing rebar in the concrete slab will alter the electric field distribution, which leads to a variation in the output voltage. This result demonstrates that the capacitive technique has promise as a method for assessing such specimens.

From the results, it can be concluded that the electric field behaviour generated by the coplanar capacitive probe is different from the test materials that are sound samples or a sample with a rebar or defect. Although the electric field does not penetrate conductive materials such as a rebar, the electric field distribution is distorted by surface defects in these materials, and these defects can therefore be detected. For a concrete slab, the electric field penetrates into it, but cannot penetrate into the conducive rebar, and so terminates at its surface, which causes a variation in the electric field pattern; hence, it is detectable by the coplanar capacitive probe.

## 5. Discussion

The traditional methods all have drawbacks that reduce their efficiency due to their high risk in specific circumstances. The coplanar capacitive sensing method has certain advantages over traditional NDT techniques for the inspection of reinforced concrete structures, which is making it a promising technique to inspect such structures. For instance, it is relatively affordable and straightforward, and there is no radiation exposure in this method. Flexibility in the design of the electrodes (various shapes/sizes) based on the applications (material under test, size/shape of the specimen) is another advantage of this technique that has led to it being a popular NDT technique. In addition, performing real experiments and scanning the specimen of this technique are simple and do not need well-trained and highly skilled operators in comparison to the other method. The coplanar capacitive probe is capable of inspecting RC structures and detecting rebars and recognising their size with an adequate difference in the measured output voltage. Moreover, this technique has the potential for repeatability. The output result of the capacitive technique for detecting rebars is simple for interpretation and does not require complex data processing to achieve desired information in comparison to GPR, radiography or ultrasonic methods.

A coplanar capacitive probe provides an accurate investigation of reinforcement concrete from a depth of a few millimetres to several centimetres, which is not possible with GPR. Furthermore, the processing of a resonant frequency value output with the coplanar capacitive technique is not required and can be easily explained with the related dielectric constant of the specimen and defect. There is no need for offline calculation or calibration stages, which are needed with radar [19]. Another comparison has been performed between the coplanar capacitive sensing technique and through-transmission air-coupled ultrasonic for the inspection of a reinforcement concrete sample containing limestone aggregate. The results demonstrated that both techniques clearly indicate the position of the rebar [27]. Therefore, the coplanar capacitive sensing technique can be a promising method for inspecting such structures.

Figure 7, which is the FEM result for the real sample, is simply comparable with Figure 10, which is the experimental result. The outputs are comparable and, in both of them, the output decreases by decreasing the size of the rebar. Based on these results, the sensitivity to the diameter of the rebars is approximately 0.26 V/inch for this coplanar capacitive sensor.

However, this technique has some restrictions. The lift-off cannot be too large and it is necessary for it to be comparable to the size of the probe. It cannot be employed for detecting the inside defects of the rebar due to the limitation of the penetration of the electric field into the conductive material. In addition, the humidity of the concrete should be controlled before using this technique because it influences the dielectric properties of the structure. Despite these limitations, the coplanar capacitive technique is becoming a potential and popular NDT method due to its inherent advantages.

## 6. Conclusions

This paper demonstrates the feasibility of the coplanar capacitive sensing technique for detecting rebars embedded in a reinforcement concrete (RC) structure. This technique works based on the difference in the permittivity between different materials in the present case, which causes a variation in the output signal when scanning the coplanar probe on the surface of the specimen. The changes in the electric field pattern in the presence of a rebar in a concrete slab are illustrated by three-dimensional (3D) finite element modelling (FEM) to demonstrate the validity of the coplanar capacitive probe as an electromagnetic NDT technique to inspect the RC structure. In addition, a set of FEM simulations was performed to analyse the effect of the surface defect on a rebar embedded in a concrete structure. It was shown that the electric field cannot penetrate conductive material such as rebars due to a high conductivity, and will accumulate on the conducting surface; hence, an equipotential surface will be created on the conductor. However, a defect on the rebar surface leads to a perturbation of the electric field distribution and a variation in the output signal. Therefore, the coplanar capacitive technique can be employed to inspect such samples. The physical experiments were conducted on a concrete slab containing different standard diameters of rebars to show the capability of the coplanar capacitive sensor to inspect RC structures. The results indicate that the probe, in addition to detecting all rebars, could also identify the different diameters of rebars. Based on this, it can be concluded that the technique has the potential to be developed to detect several different features within these kinds of structures. Developing a multi-electrode coplanar capacitive sensor with variable design parameters (such as shape/size/separation distance of the electrodes) will be helpful for this kind of investigation, which will be fully studied in future work.

## Figures and Tables

**Table 1:** Summary of the numerical model specification.

Model Parameters	Setting
Width of the electrodes	9 mm
Length of the electrodes	18 mm
Separation distance between two adjacent electrodes	4.5 mm
Width of the shielding plate	58 mm
Length of the shielding plate	36 mm
Width of the specimen	80 mm
Length of the specimen	65 mm
Height of the specimen	15 mm
Width of the computational domain	110 mm
Length of the computational domain	110 mm
Height of the computational domain	110 mm
Material of the electrodes	Copper
Material of the specimen	concrete
Material of the computational domain	Air

## Data Availability

Data sharing not applicable.
